# Clinic, Endoscopic and Histological Features in Patients Treated with ICI Developing GI Toxicity: Some News and Reappraisal from a Mono-Institutional Experience

**DOI:** 10.3390/diagnostics12030685

**Published:** 2022-03-11

**Authors:** Paola Parente, Brigida Anna Maiorano, Davide Ciardiello, Francesco Cocomazzi, Sonia Carparelli, Maria Guerra, Giuseppe Ingravallo, Gerardo Cazzato, Illuminato Carosi, Evaristo Maiello, Fabrizio Bossa

**Affiliations:** 1 Pathology Unit, Fondazione IRCCS Ospedale Casa Sollievo della Sofferenza, Viale Cappuccini 1, San Giovanni Rotondo, 71013 Foggia, Italy; i.carosi@operapadrepio.it; 2 Oncology Unit, Fondazione IRCCS Ospedale Casa Sollievo della Sofferenza, San Giovanni Rotondo, 71013 Foggia, Italy; brigidamaiorano@gmail.com (B.A.M.); davideciardiello@yahoo.it (D.C.); e.maiello@operapadrepio.it (E.M.); 3 Department of Translational Medicine and Surgery, Catholic University of the Sacred Heart, 00168 Rome, Italy; 4 Oncology Unit, Department of Precision Medicine, Università degli Studi della Campania “Luigi Vanvitelli”, 80131 Naples, Italy; 5 Division of Gastroenterology, Fondazione IRCCS Ospedale Casa Sollievo della Sofferenza, Viale Cappuccini 1, San Giovanni Rotondo, 71013 Foggia, Italy; francescococomazzi@gmail.com (F.C.); s.carparelli@operapadrepio.it (S.C.); mariaguerra.mg247@gmail.com (M.G.); 6 Section of Molecular Pathology, Department of Emergency and Organ Transplantation (DETO), University of Bari “Aldo Moro”, 70124 Bari, Italy; giuseppe.ingravallo@uniba.it (G.I.); gerycazzato@hotmail.it (G.C.)

**Keywords:** immune checkpoint inhibitor, ICI, diarrhea, colitis, PD1, PD-L1, apoptotic colitis, eosinophilic colitis

## Abstract

**Background:** Immune checkpoint inhibitors (ICIs) have widened the therapeutic scenario of different solid tumors over the last ten years. Gastrointestinal (GI) adverse events (AEs), such as diarrhea and colitis, occur in up to 50% of patients treated with ICIs. **Materials and methods:** We conducted a single-center retrospective analysis in patients with solid tumors treated with ICIs in a 6-year period, from 2015 to 2021, developing GI AEs, for which an endoscopic analysis was performed, with available histological specimens or surgery. **Results:** Twenty-one patients developed GI AEs under ICIs. The median time from the start of ICIs to the onset of GI AEs was 5 months. Diarrhea was the most frequent symptom (57.2%), upper GI symptoms presented in four patients (19%), while three patients (14.3%) had no symptoms and were diagnosed occasionally. Two patients underwent surgical resection for acute abdomen. Histological findings observed in endoscopic sampling were eosinophilic-pattern gastro-enterocolitis, apoptotic damage, IBD-like features, and ischemic-like changes. Histological damage was also documented in patients with unremarkable endoscopy. **Conclusions:** Under ICI therapy, GI toxicity is an expected event. Since GIAEs can mimic a broad range of primary GI diseases, a multidisciplinary approach is advocated with upper and lower GI mucosal sampling to remodel therapy and avoid complications.

## 1. Introduction

Over the last ten years, the therapeutic scenario of many solid tumors has been reshaped by immunotherapy, particularly with immune checkpoint inhibitors (ICIs). ICIs consist of monoclonal antibodies targeting programmed death 1 (PD-1), programmed death ligand 1 (PD-L1), or cytotoxic T-lymphocyte-associated antigen 4 (CTLA4), unleashing an anti-tumor immune response: nivolumab, pembrolizumab, and cemiplimab are directed against PD1; atezolizumab, avelumab, and durvalumab target PD-L1; and ipilimumab binds CTLA4 [1,2,3,4,5,6,7,8,9,10,11,12,13,14] (Supplementary Table S1).

Gastrointestinal (GI) adverse events (AEs) occur in up to 50% of patients treated with ICIs, and affect different tracts of the GI system. Among lower GI toxicity, diarrhea and colitis represent the most frequent AEs, sometimes leading to ICI delay or discontinuation, steroids, or other immunosuppressive drugs. Anti-CTLA4 agents have been reported to be more frequently associated with GI AEs than anti-PD1/PD-L1 agents, and double ICI combinations often cause more GI AEs than single agents. Diarrhea is reported in 23–33% of patients receiving anti-CTLA4, 10–20% of patients receiving anti-PD1/PD-L1, and 40–45% of patients treated with the combination of anti-CTLA4 and anti-PD1 agents. Colitis is reported in 7–9% of patients receiving anti-CTLA4, 1–4% of patients receiving anti-PD1/PD-L1, and 26% of patients treated with the combination. Severe colitis and diarrhea occur in 8–9% of ipilimumab-treated patients, around 1% of patients receiving PD1/PD-L1 inhibitors, and 9% of patients treated with the combination. Nausea and vomiting represent some of the most common upper GI symptoms [15,16,17]. GI AEs typically occur within the first two months from the start of ICIs, notwithstanding the fact that they can develop any time, even after ICIs discontinuation [15,16,17,18,19,20]. The pathophysiology of GI AEs involves several mechanisms, including those immune-related, due to the pharmacodynamics of the drugs, as well as gut microbiome (e.g., patients treated with ipilimumab can produce antibodies against enteric flora). Genetic predisposition and underline autoimmune conditions are other risk factors [15,16,17,21].

ICI-related diarrhea usually presents as a frequent stool associated with urgency and sometimes with abdominal pain. Bloody diarrhea is far less frequent, as well as symptoms of concurrent involvement of the upper GI tract, such as nausea/vomiting. However, the progression of symptoms is sometimes very rapid and potentially life-threatening after complications such as bowel perforation, toxic megacolon, occlusion, or ileus develop [15,16,17]. The severity of diarrhea and colitis is commonly evaluated following the Common Terminology Criteria for Adverse Events (CTCAE) (Table 1) [22].

Collaboration between oncologists and gastroenterologists is fundamental in facing these patients. Despite having limitations, an early access to a flexible sigmoidoscopy must be considerate for any patients with CTCAE ≥ G2 diarrhea. In fact, endoscopic assessment is more accurate than CTCAE for assessing disease activity and outcome [18]. The finding of a deep ulcer, for example, could be associated with a steroid-refractory colitis, which requires infliximab, vedolizumab, or other immunosuppressive agents [23]. Conversely, the severity of symptoms does not necessarily correlate with the degree of inflammation as evaluated on endoscopy and histology [24].

In this context, pathologists should be able to recognize ICI-related histologic alterations in GI biopsies for the start of early treatment. Recently, the main histologic patterns of ICI-related esophagitis, gastritis, and colitis have been described. In detail, the patterns of esophageal ICI injury consist of lymphocytic inflammation and ulcerative esophagitis; gastric injuries include chronic active gastritis, lymphocytic gastritis, and focal enhancing gastritis; and duodenal injury may present as duodenitis with villous blunting and granulomas. In the large bowel biopsies, the histologic patterns were identified as active colitis, lymphocytic and collagenous colitis (microscopic colitis), chronic active colitis, increased apoptosis colitis, ischemic colitis, and non-specific inflammatory reactive changes colitis [25]. As predicted, ICI-related histologic features in GI samples are non-specific and can mimic other types of gastro-enterocolitis, including infectious etiology, inflammatory-bowel disease (IBD), graft versus host disease (GVHD), and other drug-induced gastro-enterocolitis [26]. Therefore, clinical presentation and medical history are indispensable to distinguish between ICI-related colitis and mimics.

In this study, we aimed to describe the clinical, endoscopic, and histologic characteristics of a mono-institutional cohort of patients who developed diarrhea, colitis, or other GI AEs after treatment with anti–CTLA-4 or anti–PD-1/PDL1 drugs. Furthermore, we describe peculiar clinical and histological findings with ICI therapy that have never been reported before.

## 2. Materials and Methods

We conducted a retrospective single-center study at our institution, investigating adult patients diagnosed with solid tumors who received treatment with ICIs in a 6-year period, from 2015 to 2021. Inclusion criteria were: >18-year-old patients diagnosed with solid tumors; treated with approved anti-PD1, anti-PD-L1, or anti-CTLA4 in first-line therapy or after failure of previous therapies; and the onset of diarrhea, colitis, or other signs/symptoms of GI toxicity during ICI therapy. Only patients that underwent endoscopy with biopsies or surgery during ICI treatment were admitted. Exclusion criteria were: patients with etiologies of diarrhea/GI symptoms different from ICIs (e.g., infective etiology) when endoscopic features and histologic specimens were not available.

Once the study cohort was identified, the following data were recorded for every patient: demographics, oncologic history (site of tumor, histology, age at diagnosis, administered ICI), GI AEs characteristics (time from ICIs start to GI AE onset, symptoms at diagnosis, AE grade according to CTCAE, treatments for AEs, ICI delay/interruption, resolution of AE), as well as endoscopic and histologic findings.

All information regarding human material was managed using anonymous numerical codes, and all samples were handled in compliance with the Declaration of Helsinki (https://www.wma.net/what-we-do/medical-ethics/declaration-of-helsinki/ (accessed on 23 Semptember 2021)).

### 2.1. Endoscopy

After patients’ conscious sedation, an endoscopic examination with high-definition instruments (Olympus Medical Systems, Tokyo, Japan) was performed. Esophago-gastro-duodenal endoscopy, colonoscopy/ileo-pancolonoscopy, flexible sigmoidoscopy, or both upper and lower examinations were performed, according to the oncologist request and patients’ symptoms.

Side gross visual findings as well as inflammatory patterns of the lesions were described in the endoscopic report. In the lower GI involvement, we distinguished left-sided colitis and extensive colitis, depending on the absence or presence of lesions proximal to the splenic flexure, respectively. Gross visual findings were described as mucosal ulceration (of any morphology, size, and depth), nonulcerative inflammation (erosions, erythema, exudates, loss of vascular pattern, friability, edema, mucosal hemorrhage), or normal findings. Inflammatory patterns were divided into diffuse, patchy, or segmental groups. Given the similarity between ICI-associated colitis and inflammatory bowel disease (IBD), as reported in other studies, we sometimes used the most commonly applicated IBD endoscopic score [22].

### 2.2. Histopathology

Biopsies were fixed in 10% buffered formalin, embedded in paraffin, sectioned at 3 µm thickness, and stained with hematoxylin and eosin. All histological samples were reviewed by two expert pathologists (PP and GI), and detailed histopathologic characterization was performed on each bioptic sampling. The following lesions were assessed: the extent of GI involvement (focal, patchy, or diffuse); the presence of neutrophils within lamina propria and/or crypt epithelium (cryptitis); the presence of crypt pseudo-abscesses; the mononuclear expansion of the lamina propria; the presence of basal lympho-plasmacytosis; crypt architectural distortion (crypt branching) or irregularity (glands smaller than usual) and/or glandular loss (atrophy); the presence of Paneth cell metaplasia in the distal colon beyond the splenic flexure; increased crypt epithelial cell apoptosis (>3 apoptotic figures/10 crypts); the presence of increased intraepithelial lymphocytes within surface epithelium (>20/100 enterocytes); superficial epithelial pseudo-detachment; eosinophils count >60/10 HPF [27]; and villous blunting in duodenum.

Combining these histological features, we aimed to classify our cases into the following: eosinophilic pattern gastro-enteritis; ischemic pattern gastro-enteritis; apoptotic pattern gastro-enteritis; IBD-like gastro-enteritis; and lymphocytic gastro-enteritis. The presence of cryptitis and/or pseudo-abscess also defined each patter, such as ‘active gastro-enteritis’. Finally, if more than one pattern was observed in some cases, we recorded both (or more).

Citomegalovirus (CMV) detection was performed in apoptotic and/or IBD-like pattern by the immunohistochemistry method on paraffin embedded sections.

## 3. Results

### 3.1. Patients’ Characteristics

A total of 180 patients were treated at our center between 2015 and 2021. Diarrhea or other symptoms of GI toxicity were reported in 37 cases (21%). Among them, 21 patients who underwent an endoscopic exam with biopsies at our endoscopy unit or a surgical procedure were included in our analysis. The other 16 patients referred to external center for therapeutic management. Patients’ characteristics are shown in Table 2.

There were 17 male and 4 female patients. The median age at diagnosis was 63 years (range 50–75). Melanoma was the most frequently diagnosed tumor (*n* = 10), followed by renal cell carcinoma (*n* = 4), non-small cell lung cancer (*n* = 3), squamous head and neck carcinoma (*n* = 2), urothelial carcinoma (*n* = 1), and colon cancer (*n* = 1).

Regarding the administered ICIs, anti-PD1 were employed in 16 patients. More precisely, 11 patients (52.4%) received nivolumab, 4 patients received pembrolizumab (19%), and 1 patient (4.8%) received the combination of nivolumab plus ipilimumab. The anti-PD-L1 agent atezolizumab was administered to two patients (9.5%), while two other patients (9.5%) received ipilimumab alone. Among them, ipilimumab was administered after progression to nivolumab in two cases. The median time from the start of ICIs to the occurrence of a GI AE was 5 months (range 1–24 months).

Twelve patients developed diarrhea; among them, bleeding diarrhea was reported in four cases. In two cases (9.5%), abdominal pain was reported, and was the only presentation symptom in one of them. The severity of diarrhea and colitis was graded according to the CTCAE criteria (vers. 5.0). There were three G1, three G2, six G3, and one G4 diarrhea. In two cases (9.5%), a G4 colitis was diagnosed. One patient developed acute peritonitis, and another retrieved intestinal occlusion. Upper GI symptoms developed in four cases, with dysphagia (*n* = 3) and nausea/vomiting (*n* = 1) being the most frequent. Three patients (14.2%) were occasionally diagnosed (in one case after evidence of colitis at a CT scan and in two cases after surgical resection).

The ASCO guidelines were followed for treating patients’ AEs. The majority of patients (15–71.5%) required only symptomatic therapy, such as anti-diarrheic drugs, anti-spastic agents, or analgesics. In four patients (19%), high-dose steroids were employed. Two patients recovered with steroids; however, (9.5%) biological agents (infliximab and vedolizumab, respectively) were needed for AE management in two patients, due to the worsening symptoms under steroids. Antibiotic treatment was given to two patients with CMV detection. Due to the severity of diarrhea or colitis, ICIs were delayed in three cases (14.3%) and interrupted in five patients (23.8%). Thirteen patients (61.9%) did not require any dose delay or interruption.

### 3.2. Endoscopic Findings

A total of 21 examinations were performed in 19 patients: 8 with ileo-pancolonoscopy, 4 with colonoscopy, 1 with flexible sigmoidoscopy, and 8 with gastroscopy. Two patients underwent surgical resection for acute abdomen without endoscopic investigations. Conversely, two patients underwent both the upper and lower procedure.

**Figure 1:**
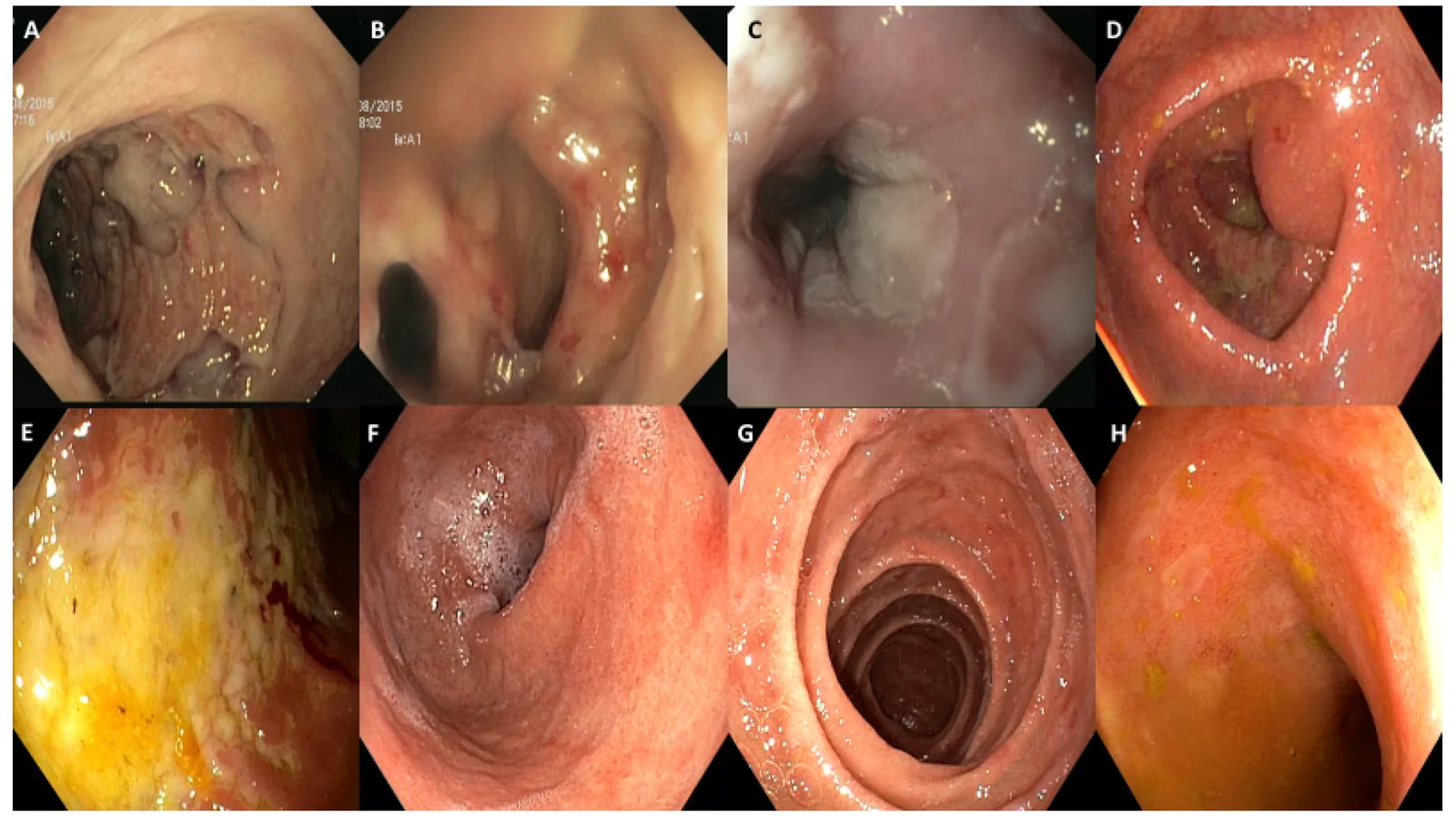
Spectrum of endoscopic findings. (**A**) Deep colonic ulceration. (**B**) Colonic fistula, demonstrated by double lumen. (**C**) Esophageal exudates not associated with Candida infection. (**D**) Colonic erythema, with friability and reduction in the vascular pattern. (**E**) Colonic exudates and mucosal hemorrhage. (**F**) Gastric erythema. (**G**) Duodenal erosions. (**H**) Ileal erosions.

In 6 of 21 endoscopies, mucosal lining results were normal. Incidentally, four colonic adenomas were detected. Endoscopic lesions were localized as follows: 1/15 (7%) in the esophagus, 5/15 (33%) in the stomach, 3/15 (20%) in the duodenum, 1/15 (7%) in the ileum, and 6/15 (40%) in the colorectal tract. For the latter, one was a pancolitis (17%), three were extensive colitis with rectal sparing (50%), one was a left-sided colitis (17%), and one was limited to rectum (17%). The gross visual findings were ulcers (*n* = 6; 40%), erosions (*n* = 9; 60%), erythema (*n* = 9; 60%), exudates (*n* = 2; 13%), loss of vascular pattern (*n* = 3; 20%), friability (*n* = 3; 20%), edema (*n* = 7; 47%), and mucosal hemorrhage (*n* = 2; 13%). In one patient, a voluminous fistula was present in the recto-sigmoidal tract. Some of these features are depicted in Figure 1. Among the six patients with colorectal involvement, inflammatory pattern was diffused in four of them (67%) or was segmental in two ones (33%). No patchy endoscopic pattern was observed.

### 3.3. Histopathological Pattern of ICI Injury

**Figure 2:**
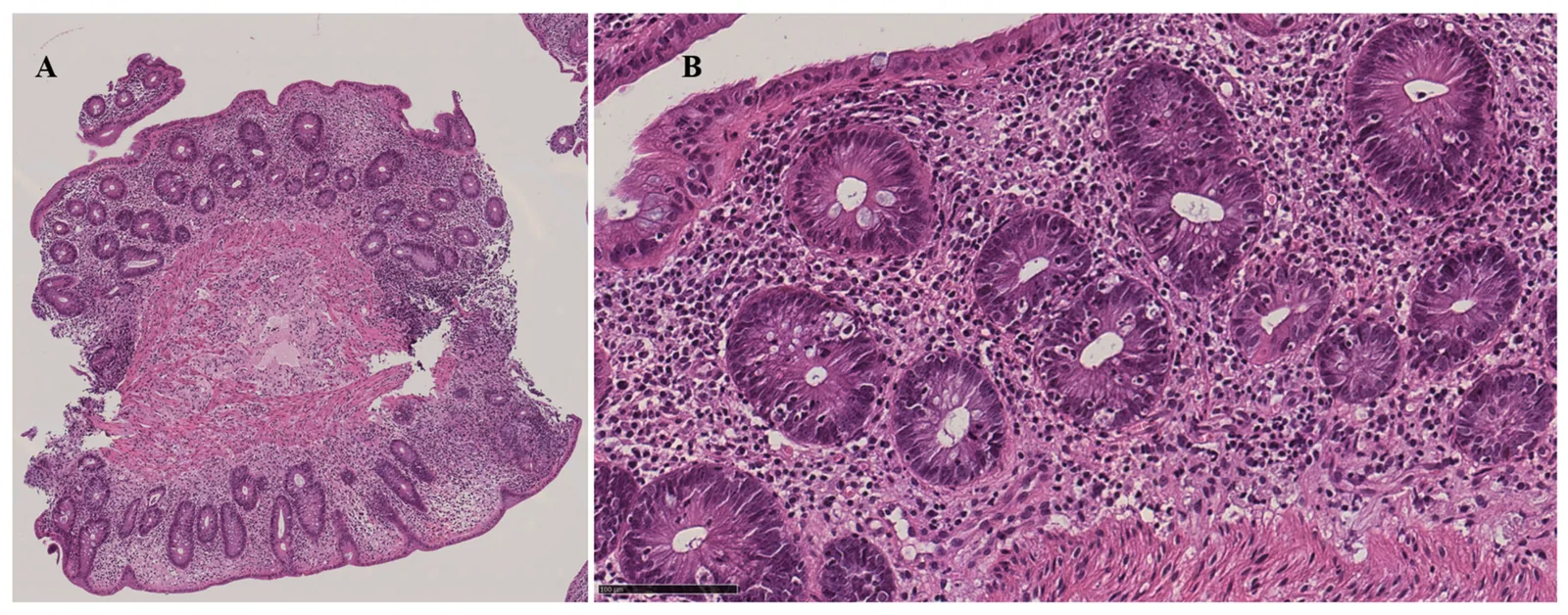
Apoptotic pattern colitis: (**A**) right colon, 9×, showing glandular atrophy and distortion; (**B**) apoptotic bodies in the glandular element, 25× (hematoxylin and eosin).

**Figure 3:**
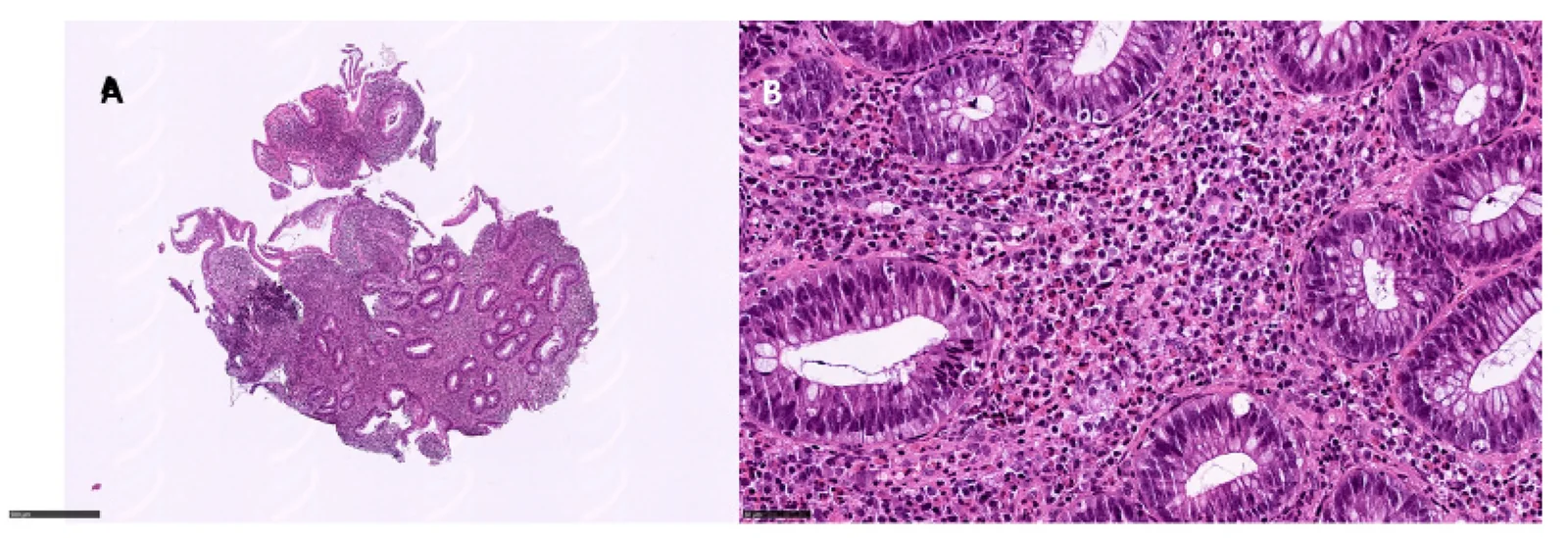
Eosinophilic pattern colitis: (**A**) left colon, 6×, showing a dense inflammatory infiltrate in the lamina propria with middle glandular distortion; (**B**) eosinophils > 60/HPF in the lamina propria (hematoxylin and eosin).

Of the 21 patients, 8 underwent ileo-pancolonoscopy with whole mucosal sampling. Among these, apoptotic combined with ischemic and eosinophilic pattern colitis was observed in two patients, an apoptotic pattern was observed in two patients (Figure 2), apoptotic combined with an IBD pattern was observed in one patient, and apoptotic combined with an ischemic pattern was observed in another patient (Table 3). Eosinophilic pattern colitis was documented in one patient (Figure 3). One patient showed only active colitis. In two patients, the villous blunting of ileal mucosa was documented. CMV was found in two patients.

Four patients underwent endoscopic intestinal polypectomy, due to endoscopic normal examen. An eosinophilic pattern was documented in three patients, while an apoptotic pattern was documented in one patient.

One patient underwent esophageal and rectal sampling after finding deep ulcers. In both histological samples, an ischemic pattern with ulcers was documented and CMV was documented by immunohistochemistry in intestinal biopsies.

Five patients underwent only gastro-esophageal endoscopy. An ischemic pattern was found in three cases, though two cases were histologically unremarkable.

**Figure 4:**
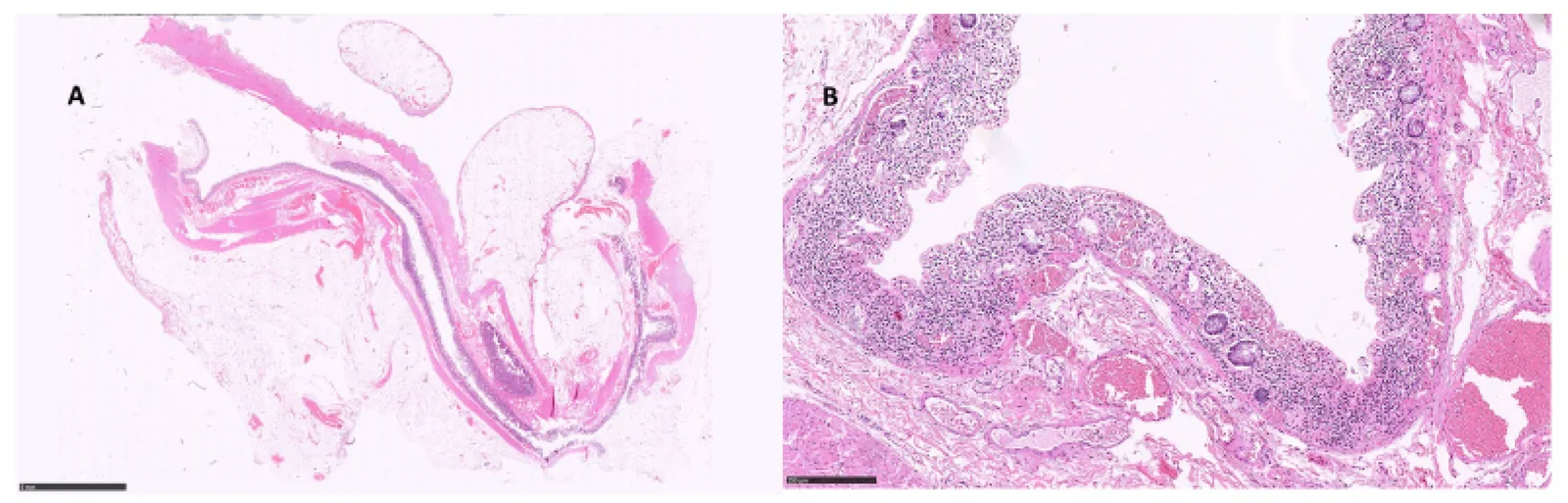
Ischemic colitis (intestinal perforation): (**A**) distal colon, showing flattened mucosa and thinned wall, 6×; (**B**) ischemic changes, consisting of extensively necrotic mucosa and neutrophilic infiltrate with blood vessels ectasia, 25× (hematoxylin and eosin).

Two patients underwent surgical resection of the left colon for acute abdomen due to an intestinal perforation; moreover, in one patient, unsuspected double synchronous colorectal cancer was found. Both patients were scheduled with PD1 therapy from 5 months and 2 months, respectively. From an histological point of view, intestinal mucosa showed an extensive and severe ischemic pattern colitis (Figure 4).

One patient underwent surgical resection of ileal segment due to a neoplastic mass; in this case, apart from neoplasia, histology was unremarkable.

## 4. Discussion

ICIs represent a groundbreaking treatment for many solid tumors, having deeply modified the treatment strategy in oncology over recent decades, with several ICIs that approved by the regulatory agencies. After binding PD1, PD-L1, or CTLA4, ICIs remove the block for the immune system, reversing neoplastic immune evasion and promoting anti-tumor response in many solid tumors. However, an off-target effect of the activation of the immune system is the development of immune-related toxicity. The risk of AEs differs between ICIs and regimens. GI tract is often involved in ICI-mediated AEs, and colitis has been reported to represent the most common cause of ipilimumab interruption [18,19,20,21,22]. With single-agent ipilimumab, diarrhea has been reported in 27.5–41% of patients (>G3 in 4.6–9.8%), while colitis has been reported in 7.6–15.5% (severe in 5.3–8.2%). Ipilimumab-related GI AEs usually occur within 2–3 months of treatment starting. Patients receiving anti-PD1 agents, such as nivolumab or pembrolizumab, develop diarrhea in 6–19% of cases and colitis in 1–4% of cases, among which severe grades occur in around 1%. The majority of GI AEs are reported within the first 6 months after the start of treatment. The toxicity profile is similar with PD-L1 antibodies. In patients treated with the combination of nivolumab and ipilimumab, diarrhea developed in 16–45% of cases and was severe in 2–9%, while colitis was diagnosed in 1–13% with severe grade in 0.5–8% [15,16,17,28,29].

Due to the onset of GI AEs, some patients undergo endoscopic examination with a wide range of mucosal injury documented in the upper and lower GI tract. Sometimes, a mucosal sampling is performed, frequently in cases with macroscopical alterations. Endoscopic findings often overlap with other primitive intestinal diseases, such as IBD (ulcerative colitis and Crohn disease). In this context, the pathologist is asked to define mucosal injury and make a histologic diagnosis. Recently, histologic features of GI mucosal tract in ICI-treated patients have been described. In a series of patients treated with PD1 inhibitors, the seven duodenal biopsies were characterized by a dense lymphoplasmacytic and eosinophilic infiltrate, villous blunting, and neutrophilic villitis. Intra-epithelial lymphocytes and apoptosis were less common findings and one case was histologically unremarkable. The six gastric samples also often demonstrated lamina propria expansion with intra-epithelial neutrophils and pseudo-abscesses; crypt apoptosis and eosinophils were observed in only one case, and one case was completely unremarkable histologically. Among five cases with ileo-colic sampling, the terminal ileum specimens showed villous blunting, increased apoptosis, lamina propria expansion, and neutrophilic villitis in three cases, whereas erosion/ulceration and increased eosinophils were documented in the other two cases. In large bowel biopsies, increased inflammation in the lamina propria and neutrophilic cryptitis was seen in the majority of cases, whereas glandular architectural distortion, neutrophilic crypt abscesses, and increased apoptosis were documented in approximately half of cases. Three patients showed an ischemic-like pattern colitis. Furthermore, thickening of the subepithelial collagen, reminiscent of collagenous colitis, was observed in one case. CMV was negative [30].

In a small case report, active colitis with apoptotic pattern was the most common documented during anti-PD-1 therapy (5/8 cases), whereas IBD pattern colitis was not observed. The distribution of the colitis was diffuse in four cases and patchy in one. In the remaining three cases, a lymphocytic colitis pattern was documented. CMV immunostaining was negative in all cases. No patients showed ischemic pattern colitis [31].

In the larger cohort of 115 patients treated with anti–CTLA-4 agent for metastatic melanoma, 85% showed an increased chronic and neutrophilic inflammatory infiltrate within the lamina propria, without chronic architectural changes in the majority of cases (61%). Apoptotic pattern was observed in a minority of cases (31%). No patients showed ischemic pattern colitis [32].

Zhang et al. described the histological features in a series of 39 patients treated with anti PD1/PDL1 and/or anti CTLA4. Peri-glandular inflammation in gastric biopsies with lymphoplasmacytic infiltrate was found in 39% of cases. Interestingly, non-necrotizing granulomas in gastric mucosa were documented in 31% of cases. Five patients showed active gastritis with neutrophilic abscesses, whereas the other five presented with a prominent reactive gastropathy pattern of injury (i.e., chemical gastritis), respectively. Thirty-six per cent showed villous blunting in duodenal biopsies and the other thirty-six showed an increase in mononuclear cells in the lamina propria. About 31 patients with concomitant intestinal sampling, acute colitis with apoptotic pattern, and lymphocytic colitis were the most common (both in 26% of cases), followed by collagenous colitis (6%) and apoptotic colitis inactive (6%). In 11 (36%) patients, the colonic mucosa was histologically unremarkable. CMV immunostaining was negative in all cases. No patients showed ischemic pattern colitis [33].

In our study, we analyzed the clinical, endoscopic, and histological findings of patients with solid tumors that were treated with ICIs in a 6-year range period in our institution. Compared to the whole cohort of treated patients, the incidence of lower GI AEs was in line with other reports (20%). Among 21 included patients, the symptomatic spectrum ranged from asymptomatic patients to the occurrence of complications, such as peritonitis, perforation, or intestinal occlusion, with diarrhea being the most frequent symptom (57.2%). Six out of twenty-one patients (28.6%) were managed with steroids or biological agents in the case of steroid-refractory diarrhea. In 8 out of 21 patients (38%), an ICI delay or even an interruption was necessary.

Our endoscopic findings were partially in line with the literature [25,34]. Unlike what was reported about the colon, we found a more frequent involvement of the right colon. These results prove that, when ICI-related colitis is suspected, a flexible sigmoidoscopy may not be sufficient. Furthermore, three of our patients had a pancolitis with rectal sparing, without any previous topical treatment. Finally, according to our knowledge, we found a case of colonic fistula associated to ICI treatment for the first time.

Histologically, we described an ischemic pattern, an apoptotic pattern, an eosinophilic pattern, and an IBD-like pattern, in isolated or combined form, in upper and lower endoscopic samples. The most frequent histologic alteration observed was an ischemic pattern (9/21 cases, 43%), an isolated form was observed in 6/21 cases (*3/16 cases of intestinal bioptic sampling and 3/5 cases of gastro-esophageal bioptic sampling*), and a combination of an apoptotic and an eosinophilic pattern was observed in 3/21 cases. The second most frequent histologic alteration was an apoptotic pattern (7/21 cases, 33%), in isolated form (3/21 cases of lower endoscopy) and combined with ischemic and eosinophilic or IBD-like pattern (4/21 cases). In 4/21 cases, we described a ‘combined’ pattern (apoptotic + ischemic + eosinophilic in 2/21 cases; apoptotic + IBD-like in 1/21 cases; apoptotic + ischemic in 1/21 cases). With respect to the literature, we found an higher percentage of ischemic alterations in GI mucosae. Interestingly, for the first time, we described the occurrence of ischemic colitis, with histologic documentation, complicated by intestinal perforation in two patients under PD1 therapy. Unfortunately, we are unable to describe hematological assessment in these patients with particular regard in the platelets’ count and proteins involved in the coagulation process; however, 43% are in high incidence and hematological data in patients with ICI therapy are lacking in all studied reported. Finally, we showed unsuspected CMV infection in 3/5 cases tested. This finding should keep in mind when steroid therapy is planned in ICI patients. The limited number of included cases is a strong limitation of our study. Furthermore, the histologic findings of mucosal damage in all patients, the unsuspected CMV detection, the previously undescribed intestinal perforation due to ischemic colitis and colonic fistula in patients with ICI therapy, and the need to indicate scheduling endoscopy with biopsies in all oncologic patients developing GI AEs are all other limitations.

Herein, we reported a mono-institutional series of GI biopsies from patients with suspected ICI-related GI AEs, drawing upon the following conclusions: (i) histologic abnormalities are often found in endoscopically normal mucosa; (ii) concurrent upper and lower GI biopsies should be obtained; (iii) ICI-associated histologic changes can mimic a broad range of primary GI diseases, including IBD and infectious enterocolitis; and (iv) in patients with intestinal comorbidities (diverticular disease, angiodysplasia), an adequate follow-up during ICI therapy is needed to avoid complications.

## 5. Conclusions

As knowledge about ICI mechanisms of action and efficacy in cancer has progressed, toxicity emerges as a potential refinement, starting from a correct and agreed diagnostic tool. GI toxicity significantly impacts the management of patients treated with ICIs for different subtypes of solid tumors; therefore, more research is warranted. In GI-symptomatic patients, an upper and lower endoscopy is indicated and, as described, an endoscopic ‘normal mucosa’ can show the histopathologic features of ICI injuries. Moreover, the ischemic mucosal damage is frequent and can lead to surgical emergencies, such as acute abdomen, which can be fatal in these patients. For this reason, biopsies of the whole GI tract are recommended for the correct assessment when ICI-related damage is clinically suspected. The recognition of histopathologic patterns of ICI-induced damage helps to form a timely and accurate pathologic diagnosis for the effective management among patients.

## Figures and Tables

**Table 1:** Common Terminology Criteria for Adverse Events (CTCAE) for diarrhea and colitis (vers. 5.0). ADL: activity of daily living.

Grade	Diarrhea	Colitis
1	Increase in stool frequency <4/day over baseline; mild increase in ostomy output compared to baseline	Asymptomatic; clinical or diagnostic observation only; intervention not indicated
2	Increase in stool frequency 4–6/day over baseline; moderate increase in ostomy output compared to baseline; limiting instrumental ADL	Abdominal pain; mucus or blood in stool
3	Increase in stool frequency >7/day over baseline; severe increase in ostomy output compared to baseline; limiting self-care ADL	Severe abdominal pain; peritoneal signs
4	Life-threatening consequences; urgent intervention indicated	Life-threatening consequences; urgent intervention indicated
5	Death	Death

**Table 2:** Characteristics of the included patients. AE: adverse event; CTLA4: cytotoxic T-lymphocyte-associated protein 4; GI: gastro-intestinal; ICI: immune checkpoint inhibitor; PD1: programmed death 1; PD-L1: programmed death-ligand 1.

Patients’ Characteristics	Values
No. of patients	21
- Male, nr. (%)	17 (81%)
- Female, nr. (%)	4 (19%)
Age at diagnosis—years, median (range)	63 (50–75)
Types of diagnosed tumors:	
- Melanoma, nr. (%)	10 (47.6%)
- Renal cell carcinoma, nr. (%)	4 (19%)
- Non-small cell lung cancer, nr. (%)	3 (14.3%)
- Head and neck carcinoma, nr. (%)	2 (9.5%)
- Urothelial carcinoma, nr. (%)	1 (4.8%)
- Colon cancer, nr. (%)	1 (4.8%)
Administered ICIs:	
- Nivolumab, nr. (%)	11 (57.2%)
- Pembrolizumab, nr. (%)	4 (19%)
- Atezolizumab, nr. (%)	2 (9.5%)
- Ipilimumab, nr. (%)	3 (14.3%)
- Nivolumab + Ipilimumab, nr. (%)	1 (4.8%)
ICI target:	
- PD1, nr. (%)	15 (71.4%)
- PD-L1, nr. (%)	2 (9.5%)
- CTLA4, nr. (%)	3 (14.3%)
- PD1 + CTLA4, nr. (%)	1 (4.8%)
Symptoms of GI AEs:	
- Diarrhea, nr. (%)	12 (57.2%)
- Bleeding diarrhea, nr. (%)	4 (19%)
- Abdominal pain, nr. (%)	3 (14.3%)
- Dysphagia, nr. (%)	3 (14.3%)
- Nausea/vomiting, nr. (%)	1 (4.8%)
- Acute abdomen, nr. (%)	2 (9.5%)
- No symptoms, nr. (%)	3 (14.3%)
Grade of reported GI AEs:	
- G1 diarrhea, nr. (%)	3 (14.3%)
- G2 diarrhea, nr. (%)	1 (33.3%)
- G3 diarrhea, nr. (%)	6 (28.7%)
- G4 diarrhea, nr. (%)	1 (4.8%)
- G4 colitis, nr. (%)	2 (9.5%)
Time from ICIs start to GI AE onset—months, median (range)	5 (1–24)
AEs management:	
- Steroids, nr. (%)	4 (19%)
- Biological agents, nr. (%)	2 (9.5%)
- Symptomatic drugs, nr. (%)	15 (71.5%)
- Antibiotics, nr. (%)	2 (9.5%)
Action taken with ICIs:	
- Delayed, nr. (%)	3 (14.3%)
- Interrupted, nr. (%)	5 (23.8%)
- None, nr. (%)	13 (61.9%)

**Table 3:** Histological pattern observed in ICI patients. GI: gastro-intestinal.

Histological Pattern	Upper GI Tract	Lower GI Tract	Total	%
Ischemic pattern	3/21	3/21	6/21	28.5%
Ischemic + apoptotic + eosinophilic pattern	0	2/21	2/21	9.5%
Ischemic + apoptotic pattern	0	1/21	1/21	4.7%
Apoptotic pattern	0	3/21	3/21	21.35%
Apoptotic + IBD-like pattern	0	1/21	1/21	4.7%
Eosinophilic pattern	0	4/21	4/21	5.2%
Active colitis	0	1/21	1/21	4.7%
Normal mucosa	2/21	1/21	3/21	21.35%

## Data Availability

Not applicable.

## Supplementary Materials

The following supporting information can be downloaded at: https://www.mdpi.com/article/10.3390/diagnostics12030685/s1, Table S1: Immune checkpoint inhibitors used in clinical practice for solid tumors.
