# Correction: Parente et al. Clinic, Endoscopic and Histological Features in Patients Treated with ICI Developing GI Toxicity: Some News and Reappraisal from a Mono-Institutional Experience. *Diagnostics* 2022, *12*, 685

**DOI:** 10.3390/diagnostics16152372

**Published:** 2026-07-28

**Authors:** Paola Parente, Brigida Anna Maiorano, Davide Ciardiello, Francesco Cocomazzi, Sonia Carparelli, Maria Guerra, Giuseppe Ingravallo, Gerardo Cazzato, Illuminato Carosi, Evaristo Maiello, Fabrizio Bossa

**Affiliations:** 1 Pathology Unit, Fondazione IRCCS Ospedale Casa Sollievo della Sofferenza, Viale Cappuccini 1, San Giovanni Rotondo, 71013 Foggia, Italy; i.carosi@operapadrepio.it; 2 Oncology Unit, Fondazione IRCCS Ospedale Casa Sollievo della Sofferenza, San Giovanni Rotondo, 71013 Foggia, Italy; brigidamaiorano@gmail.com (B.A.M.); davideciardiello@yahoo.it (D.C.); e.maiello@operapadrepio.it (E.M.); 3 Department of Translational Medicine and Surgery, Catholic University of the Sacred Heart, 00168 Rome, Italy; 4 Oncology Unit, Department of Precision Medicine, Università degli Studi della Campania “Luigi Vanvitelli”, 80131 Naples, Italy; 5 Division of Gastroenterology, Fondazione IRCCS Ospedale Casa Sollievo della Sofferenza, Viale Cappuccini 1, San Giovanni Rotondo, 71013 Foggia, Italy; francescococomazzi@gmail.com (F.C.); s.carparelli@operapadrepio.it (S.C.); mariaguerra.mg247@gmail.com (M.G.); 6 Section of Molecular Pathology, Department of Emergency and Organ Transplantation (DETO), University of Bari “Aldo Moro”, 70124 Bari, Italy; giuseppe.ingravallo@uniba.it (G.I.); gerycazzato@hotmail.it (G.C.)

In the original publication [1], there was a mistake in Figure 2A,B as published. The corrected Figure 2A,B appears below. The authors state that the scientific conclusions are unaffected. This correction was approved by the Academic Editor. The original publication has also been updated.

## Figures and Tables

**Figure 2:**
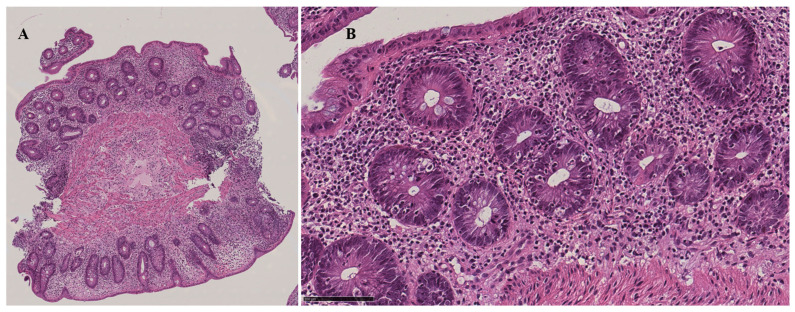
Apoptotic pattern colitis: (**A**) right colon, 9×, showing glandular atrophy and distortion; (**B**) apoptotic bodies in the glandular element, 25× (hematoxylin and eosin).
