# Supplementary material for: Clinic, Endoscopic and Histological Features in Patients Treated with ICI Developing GI Toxicity: Some News and Reappraisal from a Mono-Institutional Experience
Source: Diagnostics (Basel). 2022 Mar 11;12(3):685. doi: 10.3390/diagnostics12030685 (PMC8947154; doi:10.3390/diagnostics12030685)
Supplement: Supplementary file 1 [file diagnostics-12-00685-s001.zip › diagnostics-1599233-supplementary.pdf]

Table S1. Immune checkpoint inhibitors used in clinical practice for solid tumors.

ICI	Target	Indications
Single agents		
Ipilimumab	CTLA4	Melanoma (metastatic)
Pembrolizumab	PD1	Melanoma (metastatic, adjuvant)<br>Non-small cell lung cancer (metastatic, PD-L1 >50% for first line, PD-L1 >1% for pre-treated patients)<br>Urothelial carcinoma (metastatic, pre-treated – PD-L1>10 platinum-unfit for first line)<br>Head and neck squamous cancer (metastatic)<br>MSI-H solid tumors
Nivolumab	PD1	Melanoma (metastatic, adjuvant)<br>Non-small cell lung cancer (metastatic, pre-treated)<br>Renal cell carcinoma (metastatic, pre-treated)<br>Urothelial carcinoma (metastatic, pre-treated)<br>Head and neck squamous cancer (metastatic, pre-treated)<br>Esophageal carcinoma (adjuvant, metastatic)<br>Gastric adenocarcinoma (metastatic)
Cemiplimab	PD1	Cutaneous squamous cell carcinoma
Atezolizumab	PD-L1	Non-small cell lung cancer (metastatic, PD-L1 >50% for first-line, or pre-treated)<br>Urothelial carcinoma (metastatic, pre-treated – PD-L1>5% platinum-unfit for first line)
Avelumab	PD-L1	Merkel cell carcinoma (metastatic)<br>Urothelial carcinoma (metastatic, pre-treated or maintenance after platinum-based 1st line CT)
Durvalumab	PD-L1	Non-small cell lung cancer (stage III, PD-L1 >1% stable to CT+RT)
Combinations		
Nivolumab + Ipilimumab	PD1 + CTLA4	Metastatic melanoma<br>Non-small cell lung cancer (1st line)<br>Renal cell carcinoma (intermediate-poor risk, 1st line)<br>Pleural mesothelioma (metastatic)

		Colo-rectal cancer (MSI-H, pre-treated)
Durvalumab + CT	PD-L1 + CT	Small cell lung cancer (extended stage, 1st line)
Atezolizumab + bevacizumab	PD-L1 + anti-VEGF	Hepatocellular carcinoma (metastatic, 1st line)
Pembrolizumab + CT	PD1 + CT	Head and neck squamous cancer (metastatic, 1st line)
Pembrolizumab + axitinib	PD1 + TKI	Renal cell carcinoma (metastatic, 1st line)
Pembrolizumab + lenvatinib	PD1 + TKI	Renal cell carcinoma (metastatic, 1st line)<br>Endometrial carcinoma (metastatic, pre-treated)
Avelumab + axitinib	PD-L1 (+ TKI)	Renal cell carcinoma (metastatic, 1st line)
Atezolizumab + CT	PD-L1 + CT	Small cell lung cancer (extended stage, 1st line)<br>Triple-negative breast cancer (metastatic, 1st line, PD-L1 >1%)

CT: chemotherapy; CTLA4: cytotoxic T-lympchocyte-associated antigen 4; EMA: European Medical Agency; FDA: Food and Drug Administration; MSI-H: microsatellite instability-high; PD1: programmed death 1; PD-L1: programmed death-ligand 1; RT: radiotherapy; TKI: tyrosine kinase inhibitor; VEGF: vascular endothelial growth factor.
